# Projected Effect of Increased Active Travel in German Urban Regions on the Risk of Type 2 Diabetes

**DOI:** 10.1371/journal.pone.0122145

**Published:** 2015-04-07

**Authors:** Ralph Brinks, Annika Hoyer, Oliver Kuss, Wolfgang Rathmann

**Affiliations:** German Diabetes Center, Institute of Biometry and Epidemiology, Duesseldorf, Germany; Leibniz Institute for Prevention Research and Epidemiology (BIPS), GERMANY

## Abstract

**Background:**

Future transportation policy is likely to reduce emissions in the cities and urban regions by strengthening active travel. Increased walking and cycling are known to have positive effects on health outcomes. This work estimates effects of increased active travel on type 2 diabetes in Germany, where 64% of the population live in urban regions.

**Methods:**

Based on the effect size of an increased active travel scenario reported from a recent meta-analysis, we project the change in the life time risk, the proportion of prevented cases and the change in diabetes free life time in a German birth cohort (born 1985) compared to business as usual.

**Results:**

The absolute risk reduction of developing type 2 diabetes before the age of 80 is 6.4% [95% confidence interval: 3.7-9.7%] for men and 4.7% [2.2-7.7%] for women, respectively. Compared to business as usual, the increased active travel scenario prevents 14.0% [8.1-21.2%] of the future cases of diabetes in men and 15.8% [9.3-23.1%] in women. Diabetes free survival increases by 1.7 [1.0-2.7] years in men and 1.4 [0.6-2.3] in women.

**Conclusions:**

Our projection predicts a substantial impact of increased active travel on the future burden of type 2 diabetes. The most striking effect may be seen in the number of prevented cases. In all urban regions with an increased active travel transport policy, about one out of seven male and one out of six female cases can be prevented.

## Introduction

The recent report of the Intergovernmental Panel on Climate Change (IPCC) underlines the enormous role the transport sector has with respect to global resource use and pollution [1]. In 2010, 27% of the final energy use and 14% of the global anthropogenic greenhouse gases emissions were attributed to the transport sector. Furthermore, greenhouse gas emissions are projected to double by 2050 [1], p. 21. With a view to climate protection goals, future transport policy is likely to reduce emissions in the cities by a variety of political measures: restrictions on private motor vehicles, road-usage charges, and strengthening active travel for short distance journeys. In the climate change mitigation scenarios, the IPCC recommends investment in public transport systems and “prioritizing infrastructure for pedestrians and integrating non-motorized and transit services” [1], p. 22. Besides from reducing emissions, a change of the transport policy by promoting walking and cycling are known to have positive effects on many health outcomes. Woodcock et al found more than 7000 and 12000 saved disability adjusted life years (DALYs) per year per million population after realisation of an active travel policy for London and Delhi, respectively [2]. For this seminal work, the evidence on the effects of moderate-intensity physical activity on the incidence of severe health conditions stemmed from a systematic review [3]. Later, Jarrett et al. projected the impact of such a policy on the health services budget in England and Wales. Over a period of 20 years, increased active travel was estimated to save roughly 17 billion British pounds for the National Health Service [4].

Based on national population survey data and demographic projections for Germany, we previously predicted the enormous burden of type 2 diabetes in terms of number of cases and expenditures of health insurances for the next decades [5, 6]. Recent data confirm the forecasted upward trend [7]. One of the effects modelled by Jarrett et al refers to lowering incidence of type 2 diabetes [4]. In Germany, 64% of the population live in urban regions [8] and the proportion of the German urban population is projected to increase in the next 15 years [9]. Moreover, from the estimated 7.6 million persons with diabetes in Germany, more than 5.8 million (77%) live in urban settings [10]. Therefore, we have estimated the magnitude of possible effects of an increased active travel policy on the risk of type 2 diabetes in Germany. Germany is the country with the most inhabitants in the European Community, and comparing worldwide numbers of persons with diabetes it has the country rank 8 [10].

## Methods

Based on German epidemiological data about type 2 diabetes and the effect of increased physical activity reported in [2], we compare two scenarios: business as usual (BAU) and an increased active travel (IAT). We consider three important outcomes: (i) the difference in the life time risk of diabetes, (ii) the proportion of prevented cases and (iii) the change in diabetes free life time in a German birth cohort. The comparison between the scenarios is based on a recently developed analytical relation between the prevalence and the incidence in terms of a differential equation [11]. The differential equation allows us to calculate the prevalence of a chronic condition if its incidence, the relative mortality of the diseased persons and the mortality of the whole population (general mortality) are known.

The age- and sex-specific incidence of type 2 diabetes has been assessed in the German KORA S4 / F4 cohort study [5]. The mortality of the general population stems from the official population projection of the German Federal Statistical Office. The relative mortality risk of the diabetic population has also been taken from the KORA study. Since KORA has a limited age range, we used data of the National Danish Diabetes Register to extrapolate the incidence and mortality rates to the age groups below 45 years and above 74 years of age. As in [6] we used a proportional hazards approach. In the BAU scenario, we suppose the incidence of type 2 diabetes as input for the differential equation is as surveyed in the KORA study. The IAT scenario assumes a relative risk reduction of -0.19 [95% CI -0.27–-0.11] in the incidence of diabetes, which corresponds to an additional 2.5 hour moderate-intensity physical activity per week [2, 4]. The diabetes free life time has been calculated by Sullivan’s Method [12].

The birth cohort (born in 1985) has been chosen such that all effects of urban town building and transport policies in the next five years may have an impact on the onset of type 2 diabetes. We assume that type 2 diabetes does not occur before the age of 35, because of the very low incidence in this age group in the general population in Germany. All calculations have been performed for men and women separately. Confidence bounds are calculated by a simulation study based on 5000 random drawings from the reported distributions of the input parameters (incidence, mortality, relative risk reduction) followed by the application of a bootstrap method. Calculations have been performed with the statistical software R version 3.0.1 (The R Foundation of Scientific Computing).

### Illness-death model

**Fig 1:**
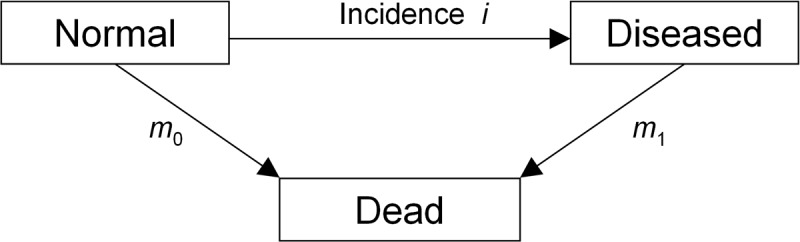
Illness-death model. Each individual in the population is in one of the three states. The transition rates *i*, *m*_0_, and *m*_1_ between the states may depend on calender time *t*, and age *a*.

For the calculation of the outcome parameters we consider an illness-death model consisting of the three states *Normal*, i.e. healthy with respect to the chronic disease under consideration, *Diseased*, and *Dead*, [13]. Henceforth, the transition rates between the states are denoted with the symbols as in Fig 1: incidence *i*, and mortality rates *m*_0_ and *m*_1_. In general, the transition rates depend on calendar time *t* (also known as *period*) and on age *a*.

In [11] we have derived a partial differential equation (PDE), which describes the relation between the age-specific prevalence *p* of the disease and the transition rates in Fig 1. The prevalence *p*(*t*, *a*) is the number of diseased persons aged *a* ≥ 0 at period *t* over the number of living persons aged *a* at *t*. With these notations, the PDE is

$$\begin{array}{c} (\frac{\partial }{\partial a}+\frac{\partial }{\partial t})\;p=\left(1-p\right)\;[i-p(m_{1}-m_{0})]. \end{array}$$ (1)

If the transition rates *i*, *m*_0_, and *m*_1_ are smooth, and we just consider diseases contracted after birth, i.e. *p*(*t*, 0) = 0 for all *t*, then the PDE (1) has a unique solution, which can be computed with standard numerical procedures.

Since we are using the relative mortality risk $R(t,a)=\frac{m_{1}(t,a)}{m_{0}(t,a)}$ and the general mortality *m* = *p* *m*_1_ + (1 − *p*)*m*_0_, the PDE (1) becomes

$$\begin{array}{c} (\frac{\partial }{\partial a}+\frac{\partial }{\partial t})\;p=\left(1-p\right)\;[i-m\;\frac{p\;(R-1)}{1+p\;(R-1)}]. \end{array}$$ (2)

Note that the fraction inside the square brackets is the *population attributable fraction*.

We consider different age- and sex-specific rates, prevalences and relative risks. For this, the superscript ℓ denotes the scenario

$$\begin{array}{c} \ell =\{\begin{array}{cc} 0 & \text{for the business as usual scenario},\\ 1 & \text{for the increased active travel scenario}; \end{array} \end{array}$$

and the superscript *s* denotes the sex

$$\begin{array}{c} s=\{\begin{array}{cc} 1 & \text{for males},\\ 2 & \text{for females}. \end{array} \end{array}$$

Then, Eq (2) reads as

$$\begin{array}{c} (\frac{\partial }{\partial a}+\frac{\partial }{\partial t})p^{\left(\ell ,s\right)}=(1-p^{\left(\ell ,s\right)})[i^{\left(\ell ,s\right)}-m^{\left(s\right)}\;\frac{p^{\left(\ell ,s\right)}\;(R^{\left(s\right)}-1)}{1+p^{\left(\ell ,s\right)}\;(R^{\left(s\right)}-1)}], \end{array}$$ (3)

for ℓ = 0, 1 and *s* = 1, 2.

### Calculation of the life time risk

The risk of contracting the chronic disease in the birth cohort (born at *t*_0_) before age *a* (conditional on survival until *a*) is

$$\begin{array}{c} P_{t_{0}}^{\left(\ell ,s\right)}\left(a\right)=\int_{0}^{a}i^{\left(\ell ,s\right)}\left(t_{0}+\tau ,\tau \right)\;\exp(-\int_{0}^{\tau }i^{\left(\ell ,s\right)}\left(t_{0}+u,u\right)+m_{0}^{\left(s\right)}\left(t_{0}+u,u\right)\;du)d\tau . \end{array}$$ (4)

In Eq (4) the mortality $m_{0}^{\left(s\right)}$ is expressed as $m_{0}^{\left(s\right)}=\frac{m^{\left(s\right)}}{1+p^{\left(\ell ,s\right)}\;(R^{\left(s\right)}-1)}.$

### Prevented cases

For a birth cohort born at *t*_0_, let $S_{t_{0}}^{\left(s\right)},\;s=1,2,$ denote the survival function:

$$\begin{array}{c} S_{t_{0}}^{\left(s\right)}\left(a\right)=\exp(-\int_{0}^{a}m^{\left(s\right)}\left(t_{0}+u,u\right)\;du). \end{array}$$

The (sex-specific) number of persons in the birth cohort who contract diabetes is

$$\begin{array}{c} C_{t_{0}}^{\left(s\right)}=N_{0}^{\left(s\right)}\;\int_{0}^{\infty }i^{\left(\ell ,s\right)}\left(t_{0}+\tau ,\tau \right)\;(1-p^{\left(\ell ,s\right)}\left(t_{0}+\tau ,\tau \right))\;S_{t_{0}}\left(\tau \right)\;d\tau , \end{array}$$

where $N_{0}^{\left(s\right)},\;s=1,2,$ is the number of cohort members born at *t*_0_. Thus, the sex-specific number of prevented cases is

$$\begin{array}{ccc} \Delta C^{\left(s\right)} & = & N_{0}^{\left(s\right)}\;\int_{0}^{\infty }\{i^{\left(0,s\right)}\left(t_{0}+\tau ,\tau \right)(1-p^{\left(0,s\right)}\left(t_{0}+\tau ,\tau \right))\\ & & -\;i^{\left(1,s\right)}\left(t_{0}+\tau ,\tau \right)(1-p^{\left(1,s\right)}\left(t_{0}+\tau ,\tau \right))\}\;S_{t_{0}}^{\left(s\right)}\left(\tau \right)\;d\tau . \end{array}$$ (5)

### Diabetes free life time

The disease-free life expectancy at age *a* is given by Sullivan’s equation [14]:

$$\begin{array}{c} e_{DF,t_{0}}^{\left(s\right)}\left(a\right)=\frac{1}{S_{t_{0}}^{\left(s\right)}\left(a\right)}\int_{a}^{\infty }(1-p^{\left(\ell ,s\right)}\left(t_{0}+u,u\right))\;S_{t_{0}}^{\left(s\right)}\left(u\right)\;du. \end{array}$$ (6)

It has been shown that Eq (6) describes an unbiased and consistent estimator for the disease free life time [12].

For type 2 diabetes, we assume *i*(*t*, *a*) = 0 for all *a* ≤ 35 and all *t*, which implies that the difference $\Delta e_{DF,t_{0}}^{\left(s\right)}$ in the disease free survival time between the two scenarios can be calculated as

$$\begin{array}{ccc} \Delta e_{DF,t_{0}}^{\left(s\right)} & = & \frac{1}{S_{t_{0}}^{\left(s\right)}\left(35\right)}\int_{35}^{\infty }(1-p^{\left(1,s\right)}\left(t_{0}+u,u\right))\;S_{t_{0}}^{\left(s\right)}\left(u\right)\;du\\ & & -\;\frac{1}{S_{t_{0}}^{\left(s\right)}\left(35\right)}\int_{35}^{\infty }(1-p^{\left(0,s\right)}\left(t_{0}+u,u\right))\;S_{t_{0}}^{\left(s\right)}\left(u\right)\;du. \end{array}$$ (7)

### Addressing uncertainty

We chose a multidimensional probabilistic setting to cope with the uncertainty in the input parameters. The key idea is to randomly sample from the distributions of input parameters (i.e., incidence, mortality of the diseased and relative risk reduction by the intervention), combine the different input parameters and calculate the outcomes (i.e, risk reduction, prevented cases, gain in disease-free life time). If we denote a set of combined input parameters by *X*, the outcomes by *Y* and the algorithm to compute *Y* from *X* by *η*, our uncertainty analysis has the form *Y* = *η*(*X*). As the input parameters *X* are sampled from random distributions many times, we get a sequence *X*_*k*_, *k* = 1, …, *K*, and the outcomes *Y*_*k*_ = *η*(*X*_*k*_) also follow a random distribution. The distribution of the *Y*_*k*_, *k* = 1, …, *K*, reflects the combined uncertainty in the outcomes based on the uncertainty in the input parameters. More about the method and the underlying ideas can be found in [15], which in the first part provides a review of the various methods in the field of uncertainty analysis.

Input parameters $m_{1}^{\left(s\right)},i^{\left(s\right)},p^{\left(s\right)},\;s=1,2,$ for Germany are sampled from the KORA data presented in [5]. The prevalence *p* ^(*s*)^ is used to calculate the relative mortality *R* ^(*s*)^, *s* = 1, 2, via the equation

$$\begin{array}{c} R^{\left(s\right)}=\frac{m_{1}^{\left(s\right)}\;\left(1-p^{\left(s\right)}\right)}{m^{\left(s\right)}-p^{\left(s\right)}\;m_{1}^{\left(s\right)}}. \end{array}$$

For Germany only data from a limited age range is available. We use data from Denmark to extrapolate the age range of the German data. In the epidemiological data from Denmark [16], we observe that the age course of

- the incidence of diabetes in men and women is log-parabolic, and
- the relative mortality $R=\frac{m_{1}}{m_{0}}$ for men and women is log-linear.

The idea of extrapolating the German data to age ranges not covered by the KORA study, is based on these two observations about the “shape” of the logarithms of the incidence and the relative mortality. Our algorithm uses the input data *X*_*k*_ (sampled from the German KORA data) to construct a log-parabolic age course of the incidence and a log-linear age course of the relative mortality. These *shape preserving* extrapolations for men and women are accomplished by the additional assumption that the log-parabola of the incidence and the log-line of *R* in Denmark and Germany differ by a constant amount. As adding a constant in the logarithmic scale corresponds to a (constant) multiplicative factor in the non-logarithmic scale, for the incidence rate this is a proportional hazard assumption: *i*_Germany_(*a*) = *β* *i*_Denmark_(*a*), for all *a*. Similarly, we obtain the assumption *R*_Germany_ = *γ* *R*_Denmark_. Note that we do not include a calendar time dependency (i.e. a period effect) in the incidence or relative mortality.

Having the methods of calculating the outcomes at hand, we can sum up the considerations about dealing with uncertainty and formulate the simulation setup as in the Algorithm shown in Table 1.

**Table 1:** Resampling algorithm.

**for** k = 1 **to** K
**for** s = 1 **to** 2
sample $m_{1}^{\left(s\right)}$, *i* ^(*s*)^, *p* ^(*s*)^ from the KORA data at ages *a* = 60 and *a* = 70
make log-parabola fit for *i* ^(0,*s*)^
make log-linear fit for *R* ^(*s*)^
sample relative risk reduction *h* and set *i* ^(1,*s*)^ ← (1 − *h*)*i* ^(0,*s*)^
calculate *p* ^(ℓ,*s*)^, ℓ = 0, 1, by solving the PDE (3)
calculate the outcomes *y*_*k*_ by applying Eqs (4) and (5), (7)
**end**
**end**

The algorithm simulates the effects of the increased active travel scenario compared to business as usual scenario.

After the calculation of the random distributions of the outcome parameters *Y*_*k*_, *k* = 1, …, *K*, we apply the *BC*_*a*_ bootstrap algorithm with 5000 replicates to calculate the 95% confidence intervals [17].

## Results

**Fig 2:**
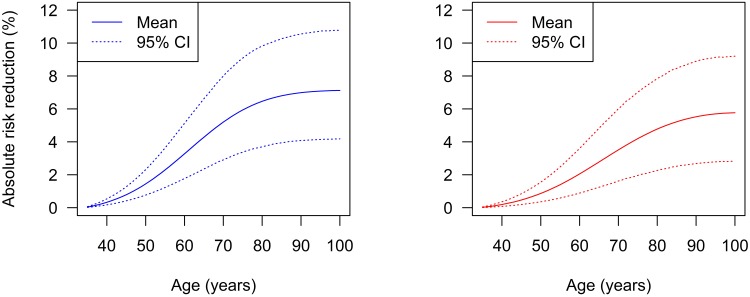
Absolute risk reduction of developing diabetes until a certain age. The absolute risk reduction (in %, solid lines) for men (left) and women (right) are shown with 95% confidence intervals (dashed lines).

Depending on the scenario, a person from the cohort has the risk of developing type 2 diabetes before a specific age as shown in Table 2. For example, in the BAU scenario the risk of a male subject contracting diabetes before 90 years of age is nearly 50%, in the IAT scenario it is about 43%. Thus, the absolute risk reduction (ARR) that is reached by implementing the IAT policy is 7%. Fig 2 shows the age-specific absolute risk reduction between the IAT and the BAU scenarios for men and women with 95% confidence bounds.

**Table 2:** Risk and risk reduction.

Age (years)	Men			Women		
	Risk (BAU)	Risk (IAT)	ARR	Risk (BAU)	Risk (IAT)	ARR
40	1.39 [0.91-1.87]	1.13 [0.73-1.54]	0.26 [0.14-0.43]	0.83 [0.39-1.28]	0.67 [0.32-1.05]	0.16 [0.07-0.29]
50	7.1 [4.7-9.4]	5.7 [3.8-7.8]	1.3 [0.7-2.1]	4.2 [2.0-6.4]	3.4 [1.6-5.3]	0.8 [0.3-1.4]
60	17.5 [11.9-23.0]	14.5 [9.6-19.3]	3.1 [1.7-4.8]	10.6 [5.1-15.9]	8.7 [4.2-13.3]	1.9 [0.8-3.4]
70	31.1 [21.8-39.6]	26.0 [17.8-34.0]	5.0 [2.8-7.8]	19.6 [9.8-28.7]	16.2 [8.0-24.3]	3.4 [1.5-5.8]
80	43.0 [31.1-53.4]	36.6 [25.7-46.6]	6.4 [3.7-9.7]	28.6 [14.7-40.9]	23.9 [12.2-35.1]	4.7 [2.2-7.7]
90	49.9 [36.8-61.0]	42.9 [30.5-53.9]	7.0 [4.1-10.5]	34.8 [18.4-48.9]	29.4 [15.2-42.4]	5.5 [2.7-8.8]

Risk of developing type 2 diabetes in the two scenarios (BAU: business as usual, IAT: increased active travel) and absolute risk reduction (ARR) (all in %) with 95% confidence intervals.

With a view to the percentage of prevented cases, the IAT scenario prevents 14.0% [8.1-21.2%] cases of type 2 diabetes in men and 15.8% [9.3-23.1%] in women of the birth cohort (compared to the BAU scenario). This means, about every one out of seven cases in men and one out of six cases in women can be prevented by shifting from business as usual to an increased active travel.

After implementing the IAT scenario, the disease free survival time increases by 1.7 [1.0-2.7] years in men and 1.4 [0.6-2.3] years in women. These numbers correspond to an increase of 4.3% [2.3-6.9%] and 2.9% [1.2-5.2%], respectively.

## Discussion

Based on the effect size reported in [2], our projection to German diabetes rates predict a large impact of the active travel policy on the diabetes risk, the number of prevented diabetes cases and the time of disease free survival.

The most striking effect may be seen in the number of prevented cases. In all urban regions that change transport policy according to the increased active travel scenario, about one out of seven male and one out of six female cases can be prevented. From the perspective of prevention of type 2 diabetes, the change to active travel policy in the cities would be very desirable. As currently about two thirds of the German population live in urban regions, the results of this work may be considerably relevant to a huge proportion of the population. In addition, the percentage of persons living in cities is projected to increase [9].

By this work, for the first time the impact of changing the transport policy to an increased active travel scenario on type 2 diabetes in Germany is estimated. We used a generic illness-death model and could show the advantageous effects on a number of diabetes related epidemiological outcomes. Apart from diabetes, increased physical activity is known to have a positive effect on a number of important health conditions such as ischemic heart disease and dementia [4]. In this work we confined ourselves to type 2 diabetes and the effects on other health conditions remain open. Another open point is the comparison of the estimated effects with other interventions, for instance, regulations on sugar consumption [18] or tobacco control [19]. However, as the illness-death model is very generic, the effects of any intervention with known reduction of incidence can be estimated.

The main weakness of our work is the data base in Germany. Unfortunately, we do not have data about incidence of diabetes and diabetes-specific mortality in certain age ranges. Thus, we had to use Danish data to extrapolate the German data. In addition, currently we do not have data about period trends in the incidence of diabetes in Germany. To be conservative, we assumed no trend in the diabetes incidence, which is in contrast to neighbouring countries, e.g. Denmark. Hence, the reported beneficiary effects of increased active travel are likely to be underestimated. Another drawback of this work is the restriction to the urban population. In rural regions the change of a transport policy, favouring more walking and cycling, is less practical (due to longer distances and a typically lower density and frequency of public transport) and, hence, seems less likely than in urban regions.

The underlying intervention of increased active travel corresponds to additional 2.5 hours per week of moderate intensity physical activity [2]. For a person working five days a week, these 2.5 hours are equivalent to additional 15 minutes of walking or cycling on the way to work and back. For someone usually going to work by car and deciding to take public transport instead, these additional 15 minutes may possibly be reached, for example, by walking to and from the bus stop and at public transport interchanges. However, it must be noted that the effects of increased active travel are considered on the *population level*. They should not be interpreted as an isolated action for an individual to prevent diabetes. Diabetes has a variety of modifiable risk factors (e.g. nutrition-related [20, 21] or smoking [22]) and a single action is unlikely to have the full potential for primary prevention on the *individual level*.

The question arises how likely political measures in favour of increased active travel in Germany are. The first signs already point in that direction. Private vehicles not fulfilling certain emission norms are already banned from parts of German cities (low emission zones, “Umweltzonen”). Increasing public budgets are provided for building bicycle lanes. Since 2002 more than 877 million Euros from the German Federal budget were spent for bicycle related projects (without taking into account investments of the federated states and municipalities) [23]. In the period 2002 to 2008 the highest increase of traffic volume has been observed in bicycles (+17%) and pedestrians (+8%) opposed to a stagnating use of private motorised vehicles (+0%) [23], p. 8. During the same period the total number of traffic deaths decreased by 35.5%, whereas the number of traffic deaths in bicyclists and pedestrians decreased to a lower extent (21.3% and 26.3%) [24]. Thus, it can be stated that the increased volume in cycling and walking currently comes at the expense of a less reduced numbers of associated traffic deaths. A detailed discussion of the role of traffic accidents and increased active travel can be found in [2, 4].

## Supporting Information

S1 Source code for the statistical software R This zip-file contains the source code for the calculation of the results in this article (including the underlying data). (ZIP)
